# AM and DSE colonization of invasive plants in urban habitat: a study of Upper Silesia (southern Poland)

**DOI:** 10.1007/s10265-016-0802-7

**Published:** 2016-02-19

**Authors:** Ewa Gucwa-Przepióra, Damian Chmura, Kamila Sokołowska

**Affiliations:** 1 Department of Botany and Nature Protection, Faculty of Biology and Environmental Protection, University of Silesia, Jagiellonska 28, 40-032 Katowice, Poland; 2 Institute of Environmental Protection and Engineering, Faculty of Materials, Civil and Environmental Engineering, University of Bielsko-Biała, 2 Willowa Street, 43-309 Bielsko-Biała, Poland

**Keywords:** Plant-microbial interactions, Root endophytes, Species invasiveness, Biological invasion, Neophytes, Knotweed, Goldenrod

## Abstract

Interactions between invasive plants and root endophytes may contribute to the exploration of plant invasion causes. Twenty plant species of alien origin differing in invasiveness were studied in terms of status and typical structures of arbuscular mycorrhizal fungi and dark septate endophytes (DSE) in urban habitats in Silesia Upland (southern Poland). We observed that 75 % of investigated plant species were mycorrhizal. The arbuscular mycorrhiza (AM) of most plant species was of the *Arum* morphology. The nearly 100 % mycorrhizal frequency, high intensity of AM colonization within root cortex and the presence of arbuscules in all mycorrhizal plant species indicate that the investigated species are able to establish AM associations in the secondary range and urban habitats. DSE were present in all mycorrhizal and non-mycorrhizal species. The frequency of DSE was significantly lower in non-mycorrhizal group of plants, however, sclerotia of DSE were found mainly in the roots of non-mycorrhizal plant species. The group of species native to North America including three *Solidago* congeners have the highest values of all AM mycorrhization and DSE indices. Moreover, we observed that most mycorrhizal invasive species belonged to the family Asteraceae. In turn, representatives of Poaceae had the lowest values of AM mycorrhization. Nevertheless, quite high values of DSE frequency were also encountered in roots of Poaceae species. The high invasiveness of the representatives of the Asteraceae family from North America support theory that both taxonomic pattern, and the fact of root endophytes colonization contribute to invasion success. While, the taxa of *Reynoutria* also represent successful invaders but they are of Asiatic origin, non-mycorrhizal and weakly colonized by DSE fungi.

**Electronic supplementary material:**

The online version of this article (doi:10.1007/s10265-016-0802-7) contains supplementary material, which is available to authorized users.

## Introduction

Arbuscular mycorrhiza (AM) is the most ancestral and commonest type of mycorrhizal symbiosis (Brundrett 2002), in which the fungal hyphae penetrate the cortical cell wall of the host plant’s root. It is characterized by the arbuscules and vesicles formed by the aseptate, obligately symbiotic fungi of the phylum *Glomeromycota* (Schüßler et al. 2001). In this association the host plant provides the fungus with assimilates i.e. soluble carbon sources, whereas the fungus provides the host plant with an increased capacity to absorb water and nutrients from the soil. It has been discovered that invasive alien plants take advantage from mycorrhizas (Klironomos 2002; Smith and Read 2008). The feedback between alien plants and soil fungal communities may strongly contribute to species invasiveness, affecting the ability of a plant to grow, establish, invade and persist in a local habitat (Bray et al. 2003; Chmura and Gucwa-Przepióra 2012). There are many case studies demonstrating that arbuscular mycorrhiza-invasive plants feedback can be rather positive than negative when AMF also become beneficial and increase their abundance (Levine et al. 2006; Stampe and Daehler 2003; Zhang et al. 2010). It is important to determine the role of AM in species invasion. It is possible that invasive alien species benefit from arbuscular mycorrhiza or conversely, they are not encouraged by arbuscular mycorrhizal fungi (AMF) and other factors influence their invasiveness (Shah et al. 2009).

Dark-septate root endophytes (DSE) are an artificial assemblage of fungi that have darkly pigmented, septate hyphae and are frequent intracellular root associates of plants (Piercey et al. 2004). They colonize the cortical cells and intercellular regions of roots and form densely septated intracellular structures called microsclerotia (Jumpponen and Trappe 1998). In contrast to the wide knowledge of arbuscular mycorrhizal fungi, the role of DSE in the ecosystem is not clearly understood. The relationship between host plants and DSE range from symbiotic to parasitic associations (Newsham 2011). At the beginning the association of DSE with plant roots was described as being parasitic (Melin 1922; Wilcox and Wang 1987) while later studies demonstrated commensal to beneficial effects on the host plant (Addy et al. 2005; Likar and Regvar 2013). Only few studies concern DSE colonization in invasive plant species (Knapp et al. 2012). Similarly to AMF it might be possible that DSE colonization play an important role in improving alien plant healthy, especially those which are non-mycorrhizal.

The invasion of alien plants alter the local biological community structure, leading to biodiversity loss (Pimentel et al. 2001; Vitousek et al. 1996). Many previous studies concerning invasion by non-native plant species were focused on aboveground features with little attention given to belowground soil organisms (Levine et al. 2004; Pyšek and Jarošík 2005; Sala et al. 2000; Vitousek et al. 1996). A few recent studies demonstrated the role of AM in plant invasion however most of studies focused on greenhouse, pot or microcosm experiments not on field studies (Koske and Gemma 2006; Richardson et al. 2000; Štajerová et al. 2009; Stampe and Daehler 2003). Root endophytes like AMF and DSE are common colonizers of plant roots across wide range of habitats (Kauppinen et al. 2014; Mandyam and Jumpponen 2005; Smith and Read 2008). In several studies, AMF and DSE have been found to enhance plant growth, photosynthetic activity, phosphorus content, act antagonistically towards soil borne fungal pathogens, and modify the concentration of plant metabolites (Toussaint 2007; Wu et al. 2010). As an important component of soil microorganisms in terrestrial ecosystem, AMF and DSE could be key factors in the invasive plant process, not only by facilitating local adaptation or reducing environmental stress but also through their effects on plant competition (Fumanal et al. 2006; Richardson et al. 2000; Wilson et al. 2012). On the other hand, invasive plants can affect function of these fungi (Callaway et al. 2008). For instance in *Ageratina adenophor* increase of AMF was observed, whereas in European *Alliara petiolata* but in invasive range (North America) native AMF were reduced (Shah et al. 2009 and literature cited therein). Some plant invaders produce allelopathicals which disrupt the belowground competitive outcome between plants and mycorrhizal fungi. The reduction of mycorrhizal colonization caused by allelopathic invasive alien plants can indirectly have a negative impact on the native plants which benefit from mycorrhiza (Bothe et al. 2010 and the literature cited therein). The plant species which negatively affect soil mycobiota are often weakly dependent on AMF or are non-mycorrhizal. Thus revitalization of some habitats, after removal of invasive species, requires the introduction of native plants which promote AMF (Ruckli et al. 2014; Tanner and Gange 2013). Our knowledge of DSE fungi diversity and their function in ecosystems and their interactions with vascular plants is limited. Thus, impact of invasive alien species is also unknown (Knapp et al. 2012).

The general objective of this study was to answer the question whether the colonization of AM and/or DSE would enhance the plant invasion. Due to limitation of the study we wanted to answer indirectly by defining the mycorrhizal status, features and the degree of colonization of arbuscular mycorrhiza fungi and dark septate endophytes of twenty invasive and alien plant species in the Polish flora and by making comparison and relating the obtained results to the species invasiveness. We hypothesize that the study of interactions between invasive plants and root endophytes may contribute to the exploration of plant invasion causes. In literature one can find rare studies which try to relate plant traits and invasion status with AM and DSE status (Majewska et al. 2015).

The second hypothesis assumes that non-mycorrhizal species should have higher frequency of DSE and within mycorrhizal species there is competition between AMF and DSE that should be revealed by negative relationships. The third hypothesis states as follows: differences in functional diversity i.e. various plant traits among plant species can be key factor explaining vulnerability to fungi colonization. The specific goals were as follows: to examine AMF and DSE type and structures in plant species differing in invasiveness and occurring in disturbed habitats within urban zone; to analyse associations between frequency of DSE and AMF colonization both between non-mycorrhizal and mycorrhizal species and within mycorrhizal species; to relate plant traits, habitat requirements and parameters of species invasiveness with AM and DSE colonization.

## Materials and methods

### Plant material and field sampling

The material was collected from Katowice city, which is situated in the centre of The Upper Silesian Industrial Region (19°00′E, 50°15′N). We selected species which are quite common and are invasive in the study area. Majority of them are neophytes (=kenophytes) sensu Tokarska-Guzik (2005) i.e. alien species introduced after the year 1500. Two species are exceptions: *Sonchus oleraceus*—archaeophyte, post-invasive plant and *Avena fatua*, synanthropic species but native to Eurasia (Table 1). Amongst neophytes there are some of the most invasive taxa in Poland and Europe: *Reynoutria* spp, highly invasive *Solidago canadensis, S. gigantea, Impatiens parviflora, I. glandulifera* and also weakly invasive species such as *Cardaria draba, Eragrostis minor* (Tokarska-Guzik et al. 2012). In respect to invasion status (Richardson et al. 2000) in the study region (Silesian Upland), ten species are considered as transformers i.e. subset of invasive species that have clear ecosystem impact, five species are weeds i.e. those plants which grow in sites where they are not wanted, for instance—arable fields. Five species are classified as not-harmful or non-invasive (Tokarska-Guzik et al. 2010). In total, 20 plant species were collected during the flowering period in 2012. The range of a species (scale: 0–5) and abundance of population (scale: 1–5) was given after Zarzycki et al. 2002, whereas tendency (1–4) and invasiveness (scale: 1–21) was adopted after Tokarska-Guzik et al. 2010. The nomenclature of vascular plant species follows Mirek et al. 2002. All plants were collected from urban and suburban habitats i.e. wastelands, roadsides, disturbed managed forests. In total 20 sites were chosen. At each site four repetition samples were taken. In order to avoid pseudoreplication repetition samples were gathered from places away from each other. To evaluate fungi root endophytes colonization, root samples of at least five flowering plants of each species were collected from a depth of 0–20 cm for one sample. Sampling was carried out in the peak of flowering period for each taxon separately. Only well-developed, undamaged individual were taken. The list of species with some characteristics is given in Table 1, whereas detailed information about GPS coordinates and types of habitat is in attachment (Table S1, Supplementary Material). Plants were excavated in their entirety and manually cleaned of soil.

**Table 1:** The list of species with their invasion characteristics on the country and the regional scale

Family	Plant species	Invasion status	Geographical—historical status	Range	Population size	Tendency in spread	Invasiveness
		(Silesian Upland)					
*Asteraceae*	*Aster novi*-*belgii* L.	Transformer	Neophyte	3	4	4	15
	*Bidens frondosa* L.	Transformer	Neophyte	4	3	2	10
	*Conyza canadensis* (L.) CORNQ.	Weed	Neophyte	3	3	2	13
	*Erigeron annus* (L.) PERS.	Not-harmful	Neophyte	5	3	2	10
	*Galinsoga ciliata* (RAF.) S.B.BLAKE	Weed	Neophyte	3	5	4	17
	*Galinsoga parviflora* CAV.	Weed	Neophyte	4	1	1	11
	*Solidago canadensis* L.	Transformer	Neophyte	4	4	3	12
	*Solidago gigantea* AITON	Transformer	Neophyte	4	3	2	10
	*Solidago graminifolia* (L.) ELLIOTT	Non-invasive	Neophyte	5	3	2	10
	*Sonchus oleraceus* L.	Weed	Archaeophyte	4	5	3	16
*Brassicaceae*	*Cardaria draba* (L.) DESV.	Non-invasive	Neophyte	4	5	2	15
	*Diplotaxis muralis* (L.) DC.	Non-invasive	Neophyte	4	5	3	17
*Balsaminaceae*	*Impatiens glandulifera* ROYLE	Transformer, post-invasive	Neophyte	4	5	3	18
	*Impatiens parviflora* DC.	Transformer	Neophyte	3	5	4	16
*Cucurbitaceae*	*Echinocystis lobata* (MICHX.) TORR.&A.GRAY	Transformer, post-invasive	Neophyte	5	2	0	6
*Poaceae*	*Avena fatua* L.	Weed	Archaeophyte	3	1	2	2
	*Eragrostis minor* HOST	Not-harmful	Neophyte	4	3	2	8
*Polygonaceae*	*Reynoutri*a × *bohemica* CHRTEK&CHRTKOVA	Transformer, post-invasive	Neophyte	3	5	4	21
	*Reynoutria japonica* HOUTT.	Transformer, post-invasive	Neophyte	4	5	4	21
	*Reynoutria sachalinensis* (F.SCHMIDT) NAKAI	Transformer,	Neophyte	3	5	2	19

### Assessment of AMF and DSE colonization

For the estimation of mycorrhizal development, the roots were prepared according to a modified method of Phillips and Hayman (1970). After careful washing in tap water the roots were softened in 7 % KOH for 24 h and then rinsed in a few changes of water. The material was acidified in 5 % lactic acid for 24 h and then stained with 0.01 % aniline blue in lactic acid for 24 h. The entire procedure was carried out at room temperature. Root fragments approximately 1 cm long, at 30 fragments per one repetition sample, were mounted on slides in glycerol: lactic acid (1:1) and pressed using cover slides. In total 120 fragments were taken for particular species.

AMF colonization and AM morphology were identified on the basis of aseptate hyphae growing intracellularly, forming arbuscules terminally in the cortical cells (the *Arum*-type AM morphology); intracellularly with arbuscules developed on coils in the cortical cells (the *Paris*-type) or forming intermediate types (Dickson 2004).

The following parameters describing the intensity and effectiveness of the mycorrhization were recorded: mycorrhizal frequency (F %)—the ratio between root fragments colonized by AMF mycelium and the total number of root fragments analyzed, relative mycorrhizal root length (M %)—an estimate of the amount of root cortex that is mycorrhizal relative to the whole root system, intensity of colonization within individual mycorrhizal root (m %), relative arbuscular richness (A %)—arbuscule richness in the whole root system and arbuscule richness in root fragments where the arbuscules were present (a %) (Trouvelot et al. 1986). DSE colonization was identified on the basis of regularly septate hyphae, usually dark pigmented, with facultatively occurring sclerotia (Jumpponen 2001). The mycelium does not stain with aniline blue and remain brownish. The frequency of DSE mycelium (hyphae and sclerotia) occurrence in roots (FDSE %) was estimated similarly as it was calculated for the mycorrhizal frequency (Nobis et al. 2015; Zubek and Błaszkowski 2009).

### Statistical analysis

In order to compare species cluster analysis was done on the basis of mean values of mycorrhization indices i.e., F %, M %, m %, A %, and a % and FDSE %. Clustering methods such as Euclidean distance and Ward method were applied. To do this, arithmetic means of mycorrhization indices per species were calculated. The obtained clusters of species were analyzed in terms of particular AM and DSE indices. The significance of differences in FDSE % between distinguished groups was performed by the Kruskal–Wallis test followed by the Conover test for multiple comparisons, whereas AM colonization indices were tested using Wilcoxon sum rank test only within AM species. The relationship between DSE and mycorrhization by arbuscular fungi was carried out by the Spearman rank correlation analyses between FDSE % and mycorrhization indices. All samples were subjected to this analysis except for non-mycorrhizal plants (all indices of AM equal zero). To estimate whether plant traits, their habitat requirements and invasiveness have an influence on AM colonization and frequency of DSE two statistical approaches were employed. For the purpose of these analyses the following traits were used (plant traits), i.e. Grime strategy (the following strategies were used: C/CSR competitive/intermediate strategy, CR competitive ruderal, R ruderal, R/CR ruderal/competitive ruderal, SR stress ruderal), mean height of stem, and type of seed bank (no seed bank, short-term, long-term bank). As a measure of habitat associations, Ellenberg indicator values (EIVs) for moisture F, soil reaction R and trophy N were adopted. Finally, data about invasiveness of species was included. As a measure of species invasiveness the following data was included: range—expressed in 5 scale; population size (5° scale); the type of habitats colonized (3° scale) habitats invaded; dynamic tendency (5° scale) i.e. tendency in spread; and residence time (time since putative date of introduction till 2005 (Tokarska-Guzik 2005, 2012; Zarzycki et al. 2002). Since we did not have abundance data of species we used multidimensional functional diversity (FD), which does not require abundance and presence/absence data (Mouillot et al. 2013). We treated groups of species revealed after cluster analysis as “community”, in the sense of species which respond in a similar way to AM and DSE colonization. We computed distance-based functional diversity indices FD using R library FD: functional richness (FRic), functional evenness (FEve), and functional divergence (FDiv) (Villéger et al. 2008) as well as functional dispersion (FDis; Laliberté and Legendre 2010), Rao’s quadratic entropy (Q) (Botta-Dukát 2005) and the community-level weighted means of trait values (CWM; e.g. Lavorel et al. 2008). Since FD does not provide a formal statistical test for the significance of differences among communities, we applied ordination technique—Redundancy Analysis and permutation test. To that end, three RDAs redundancy analyses were performed based on two matrices. In all, RDAs first matrix contained data of F %, M %, m %, A %, and a % including non-mycorrhizal plants (values of AM colonization equal zero) and FDSE %. Contrary to cluster analysis, raw data from repetitions, instead of means, was included. In first RDA, as shown in the second table, matrix data on plant traits was employed. The second RDA was done with habitat associations and the third one with invasiveness features. In total, 999 permutations were computed to assess statistical significance of variables in the model. All statistical analyses were performed using R software (R Core Team 2015).

## Results and discussion

### Mycorrhizal studies

In this work, we present a detailed report on the mycorrhizal status, AMF colonization rate and AM morphology of 20 alien plant species in Polish flora. Arbuscular mycorrhiza were found in 15 out of 20 investigated plant species except for the roots of *Cardaria draba, Diplotaxis muralis* and all *Reynoutria* species (Table 2). The AM of 13 plant species was of the *Arum* morphology. Hyphae were observed mainly in the intercellular spaces of root cortex, forming arbuscules terminally in cortical cells. Only one species—*Bidens* *frondosa* was characterized by *Paris*-type colonization in which neighbouring cortical cells contained hyphal coils, without hyphae in the intercellular spaces. The intermediate AM colonization was found only in *Erigeron annuus* roots (Table 2). The mycorrhizal structures of all investigated plant species found to host AMF comprised arbuscules and vesicles, with the exception of *Avena fatua* and two *Impatiens* species, in which the mycorrhizal roots did not contain vesicles. Coils were encountered in only two species belonging to Asteraceae family (Table 2). In roots of all mycorrhizal plants only coarse AMF (hyphae diameter above 2 μm) were found. The fine AM endophyte (*Glomus tenue*) was not observed at all.

**Table 2:** Alien plant species AM status and type; AM and DSE structures of investigated species

	AM literature status^a^				AM	AM structures^b^			DSE
	H&H	W&Q	S	EGP	Type	A	V	C	Hyphae sclerotia
*Aster novi*-*belgii*	+	+	+	+	Arum	+	+	−	+ −
*Bidens frondosa*	nd	+	+	+	Paris	+	+	+	+ −
*Conyza canadensis*	nd	+	+	+	Arum	+	+	−	+ +
*Erigeron annus*	+	+	+	+	Intermediate	+	+	+	+ +
*Galinsoga ciliata*	+	+	+	+	Arum	+	+	−	+ −
*Galinsoga parviflora*	+	+	+	+	Arum	+	+	−	+ −
*Solidago canadensis*	nd	+	+	+	Arum	+	+	−	+ −
*Solidago gigantea*	nd	+	+	+	Arum	+	+	−	+ −
*Solidago graminifolia*	nd	+	nd	+	Arum	+	+	−	+ −
*Sonchus oleraceus*	+	+	nd	+	Arum	+	+	−	+ +
*Cardaria draba*	−	nd	nd	−	NM	−	−	−	+ +
*Diplotaxis muralis*	−	±	nd	−	NM	−	−	−	+ −
*Impatiens glandulifera*	+	±	+	+	Arum	+	−	−	+ −
*Impatiens parviflora*	+	±	+	+	Arum	+	−	−	+ −
*Echinocystis lobata*	nd	nd	+	+	Arum	+	+	−	+ −
*Avena fatua*	+	+	nd	+	Arum	+	−	−	+ −
*Eragrostis minor*	nd	nd	nd	+	Arum	+	+	−	+ −
*Reynoutri*a × *bohemica*	nd	nd	−	−	NM	−	−	−	+ +
*Reynoutria japonica*	−	nd	−	−	NM	−	−	−	+ +
*Reynoutria sachalinensis*	−	nd	−	−	NM	−	−	−	+ +

^a^ *Plus* AM present, *minus* lack of AM, *nd* no data, *H&H* Harley and Harley 1987, *W&Q* Wang and Qiu 2006, *S* Štajerová et al. 2009, *EGP* own studies

^b^ *A* arbuscules, *V* vesicles, *C* coils, *plus* present, *minus* absent

Analysis of the mycorrhizal status of investigated invasive alien plant species showed that 75 % of them were associated with AMF of the phylum Glomeromycota. In the majority of species investigated in this study, AM status has already been known. However, previous studies were based on reviews by Harley and Harley (1987) or Wang and Qiu (2006) therefore most of those plants were analyzed as native species in their natural habitat range e.g. *Solidago graminifolia* (Table 2). However, the finding of the AM in *Eragrostis minor* by the authors of this paper is the first report of the mycorrhizal status of this plant. Also, the mycorrhizal status of some neophytes evaluated in our research has already been given, but only in the Czech Republic so far (Štajerová et al. 2009) (Table 2). However, they did not give AM morphotype and level of colonization in roots cortex of those plants.

The dominance of the *Arum*-type among the plant species we studied is comparable with a previous report, where this AM morphotype was also the most common in non-native plants from India (Shah et al. 2009). Plant species identity plays a major role in determining the pattern of AMF development in roots, although AM-type may depend on fungal identity and environmental conditions (Smith and Read 2008). The dominance of the *Arum*-type among investigated invasive plant species may therefore not be incidental.

The richness of mycorrhizal structures in roots varied among different species. However average frequencies (F %) of all mycorrhizal species were very high and ranged from 86 to 100 %. Intensity of AMF colonization in root system (M %) and within individual mycorrhizal root (m %) was between 3 and 62 %. Root colonization (M %, m %) of many AM plant species was high and reached over 40 % (Table 3). All of them belong to the Asteraceae family. In contrast, low M % and m % values were observed only in two investigated grass species. The high level of AM frequency and root cortex colonization within mycorrhizal species indicates that investigated plant species are able to establish AMF associations in their new urban and suburban habitats in Silesia Upland. Another indicator of well functioning mycorrhiza of investigated alien plants is the presence of arbuscules in all plant species recognized to associate with AMF and high arbuscular richness of most species. Arbuscules are structural and functional criterion of this kind of mycorrhizal symbiosis. Both measures of root arbuscules occurrence (A % and a %) followed the same pattern as mycorrhizal colonization indices (M %, m %). The highest mycorrhizal parameters were observed in *Erigeron annuus* roots and other plant species of the Asteraceae family (Table 3). The highest value of arbuscule abundance in whole root system (A %) was about 50 % whereas arbuscule richness of the colonized root section (a %) was above 88 %. The highest values of arbuscular richness were observed in plant species of the Asteraceae family (Table 3). The lowest arbuscule occurrence was found in *Avena* *fatua* and *Eragrostis* *minor*—representatives of Poaceae family (Table 3). The latter species is a typical urban plant, which is frequently found in many Central European towns (Brandes 1995) and occurs even in harsh conditions e.g. tramlines (Sudnik-Wójcikowska and Galera 2005). In such habitats there are no favourable conditions for AMF development. Although in greenhouse cultivation the presence of AMF enhances growth of the species through increasing the weight of seedlings, it is treated as non-dependant on AMF species (Wurst et al. 2011). Generally, AMF are known as ubiquitos in grass roots in different habitats, even the harsh ones. (Gucwa-Przepióra et al. 2007; Gucwa-Przepióra and Błaszkowski 2007; Kauppinen et al. 2014). Also, previous research showed well functioning AM and high level of mycorrhizal colonization in exotic grass *Miscanthus* × *giganteus* from sites contaminated by heavy metals in Silesia Upland in Poland (Gucwa-Przepióra et al. 2010). There was a considerable difference in the mycorrhizal colonization and arbuscule abundance between native and invasive grass species in Hungary. Lower degrees of AMF colonization parameters were observed for invasive grasses than for native residents in the Hungarian semiarid grassland community (Endresz et al. 2013).

**Table 3:** AM and DSE colonization parameters of studied alien plant species

	F %	M %	m %	A %	a %	FDSE %
*Aster novi*—*belgii*	100.0 ± 0.0	48.5 ± 4.1	48.5 ± 4.1	26.0 ± 4.1	53.6 ± 6.4	50.00 ± 2.72
*Avena fatua*	86.3 ± 14.2	2.91 ± 1.6	3.0 ± 1.5	0.8 ± 1.1	21.0 ± 20.7	77.08 ± 2.63
*Bidens frondosa*	100.0 ± 0.0	15.3 ± 3.8	15.3 ± 3.8	11.4 ± 1.3	77.5 ± 12.7	40.00 ± 2.72
*Conyza canadensis*	100.0 ± 0.0	42.5 ± 2.7	42.5 ± 2.7	21.3 ± 1.4	50.0 ± 0.9	26.66 ± 7.20
*Echinocystis lobata*	100.0 ± 0.0	12.5 ± 3.6	12.5 ± 3.6	4.2 ± 1.3	33.5 ± 0.6	16.25 ± 7.25
*Eragrosis minor*	100.0 ± 0.0	6.5 ± 2.6	6.5 ± 2.6	0.8 ± 0.7	13.2 ± 10.7	16.66 ± 2.72
*Erigeron annus*	100.0 ± 0.0	53.4 ± 7.7	53.4 ± 7.7	47.9 ± 11.4	88.6 ± 9.3	19.58 ± 2.85
*Galinsoga ciliata*	100.0 ± 0.0	29.7 ± 5.0	29.7 ± 5.0	12.6 ± 1.4	43.0 ± 5.7	17.07 ± 4.78
*Galinsoga parviflora*	100.0 ± 0.0	43.6 ± 3.7	43.6 ± 3.7	25.3 ± 2.8	58.6 ± 8.8	44.16 ± 3.19
*Impatiens glandulifera*	100.0 ± 0.0	31.7 ± 11.4	31.7 ± 11.4	16.0 ± 7.6	48.4 ± 6.7	16.50 ± 0.33
*Impatiens parviflora*	100.0 ± 0.0	24.3 ± 13.3	24.3 ± 13.3	10.1 ± 8.1	33.7 ± 16.9	14.25 ± 3.28
*Solidago canadensis*	100.0 ± 0.0	62.2 ± 4.6	62.2 ± 4.6	28.8 ± 1.8	46.4 ± 0.8	16.29 ± 5.85
*Solidago gigantea*	100.0 ± 0.0	51.5 ± 7.4	51.5 ± 7.4	24.9 ± 7.4	47.2 ± 8.5	12.08 ± 1.59
*Solidago graminifolia*	100.0 ± 0.0	44.5 ± 20.5	44.5 ± 20.5	21.2 ± 11.4	45.2 ± 6.8	19.15 ± 3.19
*Sonchus oleraceus*	100.0 ± 0.0	19.3 ± 10.2	19.3 ± 10.2	11.0 ± 8.3	53.0 ± 11.5	12.91 ± 2.87
*Cardaria draba*	0	0	0	0	0	26.67 ± 7.20
*Diplotaxis muralis*	0	0	0	0	0	16.67 ± 2.72
*Reynoutri*a × *bohemica*	0	0	0	0	0	19.15 ± 3.19
*Reynoutria japonica*	0	0	0	0	0	12.87 ± 2.87
*Reynoutria sachalinensis*	0	0	0	0	0	10.0 ± 0.0

Values are mean ± SD

*F* *%* mycorrhizal frequency, *M* *%* relative mycorrhizal root length, *m* *%* intensity of colonization within individual mycorrhizal root, *A* *%* arbuscule richness in the whole root system, *a* *%* arbuscule richness in root fragments where the arbuscules were present, *FDSE* *%* DSE frequency

On the other hand, important plant families to which many invasive plant species belong, are often considered as non-mycorrhizal e.g.: *Brassicaceae*, *Polygonaceae*, *Chenopodiaceae*, *Caryophyllaceae* (Harley and Smith 1983). Our research confirmed that a group of *Reynoutria* species (*Polygonaceae*) although non-mycorrhizal was very successful in the invasion process. This result supports the hypothesis of Pringle et al. (2009) that an invasive plant is likely to be non-mycorrhizal or a facultative symbiont. Also, non-mycorrhizal plant species prefer disturbed sites such as the early stages of industrial heaps (Gucwa-Przepióra and Turnau 2001; Janos 1980) and ruderal sites (Gange et al. 1990).

It is believed that there is a taxonomic pattern in invasiveness plants. As Pyšek (1998) in a worldwide review study demonstrated, the largest families (*Poaceae*, *Asteraceae*, *Brassicaceae*) contribute most to the total number of alien species in local floras. These families are also the most species-rich taxa. However, when it comes to the pool of potentially invasive species, *Polygonaceae* and *Poaceae* are prevalent (Pyšek 1998), this was also true for representatives of those families in our study. The most successful species in terms of invasiveness derive from these taxa. The most successful families possess some properties that could be attributed to their invasiveness, but these are rather complex and can hardly be related to the invasiveness of a particular family (Pyšek 1998). Perhaps independence from arbuscular mycorrhiza or weak dependence, expressed by lower values of AM colonization, may be considered a trait that makes some plants more invasive. That means that some species can thrive in sites free from AMF in soils. They do not need root endophytes to establish, persist and finally initiate further spread and become invasive.

### DSE colonization

DSE were found in all investigated plant species both in mycorrhizal and non-mycorrhizal species (Table 3). In the case of AM species DSE were observed in the cortex together with AMF but mainly in root fragments where arbuscules were absent. The regularly septated hyphae, accompanied sporadically by sclerotia, were found in rhizodermis and outer cortical cells. The frequency of DSE occurrence in roots of most species was below 50 % or even 20 %. The exception was *Avena fatua* roots where DSE colonization was observed to be more frequent (FDSE % >70 %). In contrast to the wide knowledge of arbuscular mycorrhizal and ectomycorrhizal fungi (Smith and Read 2008), relatively little is known about the DSE fungi and their functions, although various reports of positive impacts of DSE colonization on their plant hosts have supported the view that DSE do indeed have a beneficial role for plant growth and survival (Fernando and Currah 1996; Mullen et al. 1998). Among the fungal endophytes that colonize the roots, DSE are often frequent colonists of plants even when they are growing under extreme conditions, like drought (Barrow 2003), high salinity (Sonjak et al. 2009), and metal-enriched soils (Deram et al. 2008; Likar and Regvar 2013). Some authors have suggested that DSE may assume the role of AMF especially in the case of taxa which are rarely or not colonized by AMF like *Carex* species (Haselwandter and Read 1982), *Atriplex* *canescens* (Barrow et al. 1997), *Saponaria* *officinalis* (Zubek and Błaszkowski 2009). We believe that is unlikely because in our study DSE were present in all mycorrhizal and non-mycorrhizal species and in the latter group DSE are less frequent. Thus, DSE are rather not alternative to AMF in terms of all aspects of positive symbiosis, but this requires further detailed ecophysiological research.

### Functional analysis of AM and DSE colonization

**Fig. 1:**
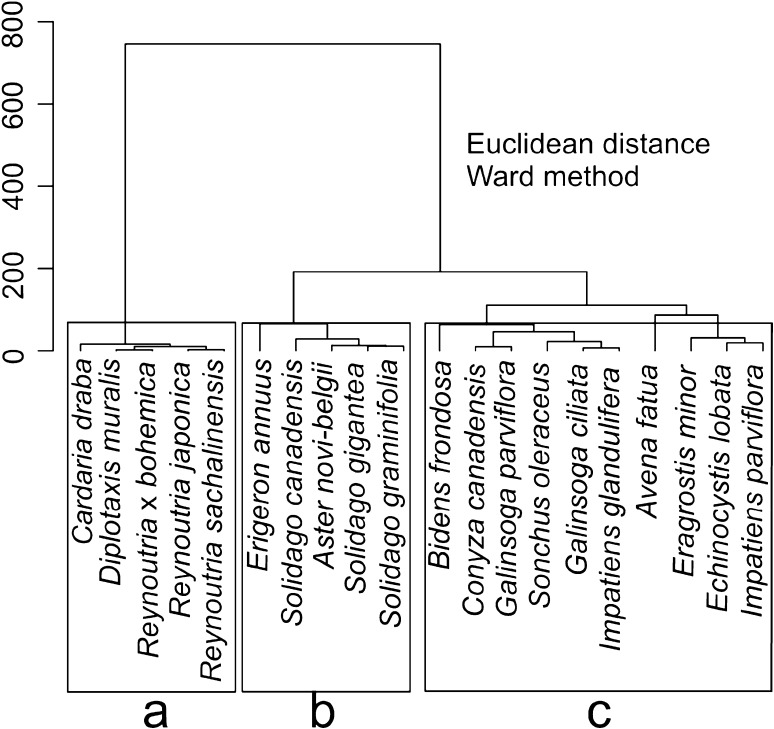
Cluster analysis of species on the basis of mean values of AM and DSE colonization indexes. **a** Non-mycorrhizal species. **b** *Aster novi*-*belgii* group. **c** *Galinsoga ciliata* group

**Fig. 2:**
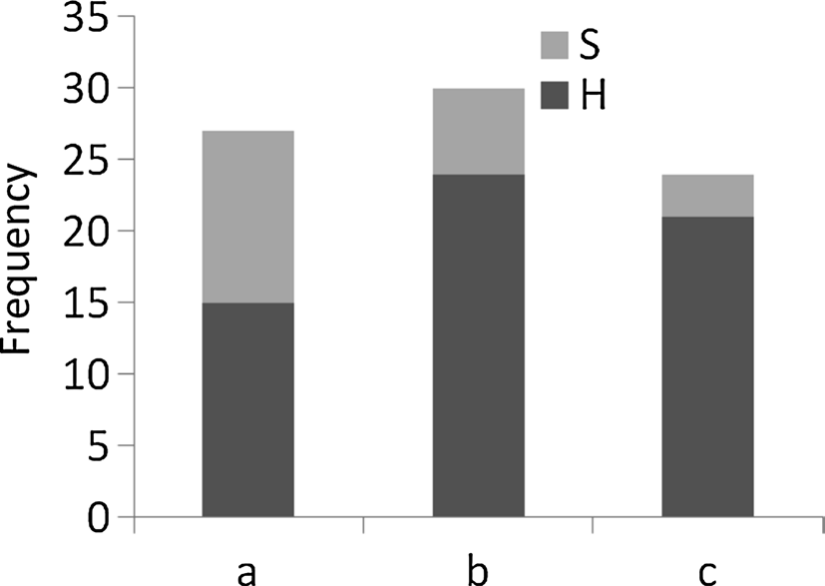
Comparison of frequency of sclerotia (*S*) and hyphae (*H*) of DSE among distinguished groups of plants. **a** Non-mycorrhizal species. **b** *Aster novi*-*belgii* group. **c** *Galinsoga ciliata* group

On the basis of AM and DSE colonization indices cluster analysis revealed three groups of plants (Fig. 1). The first group comprises non-mycorrhizal plants. The remaining two groups contained 5 and 10 species, respectively: *Aster novi*-*belgii* group and *Galinsoga ciliata* group. For instance in the same group congeneric species were found i.e. three taxa from *Solidago* genus (*Aster novi*-*belgii* group) as well as *Galinsoga ciliata* and *G. parviflora* and both *Impatiens* species in the second group. FDSE % significantly varied between the three mentioned groups (Kruskal–Wallis, Chi-squared = 16.50, p < 0.001), but it was the lowest in non-mycorrhizal plants (Table 4). As only AM + species are concerned *Aster novi*-*belgii* group has significantly higher values of M %, m %, A % and a % indices (Table 4). Moreover, in mycorrhizal species there is a weak negative correlation between AM colonization indices and frequency of DSE, however, in case of F % it turned out to be significant (rs = −0.47, p < 0.0001). Taking into account all samples frequency of hyphae vs sclerotia among clusters of plant species varied significantly; hyphae were significantly more frequent (Chi-squared = 8.702, p = 0.013) in AM plants i.e. *Aster novi*-*belgii* group followed by *Galinsoga ciliata* group (Fig. 2).

**Table 4:** Comparison of DSE frequency (FDSE %), the different letters (^a^, ^b^,^ c^) near values demonstrate significant differences after Kruskal–Wallis test (p < 0.001) followed by Conover test, among all groups of species and comparison of chosen AM colonization indices between two mycorrhizal groups (Wilcoxon sum rank test)

	Non-mycorrhizal species	*Aster novi*-*belgii* group	*Galinsoga ciliata* group
F %	–	NS 100.0 ± 0.0	96.6 ± 9.0
m, M %	–	45.2 ± 15.1**	16.2 ± 13.0
A %	–	25.9 ± 11.4**	7.0 ± 7.3
a %	–	58.4 ± 16.8***	33.4 ± 18.4
FDSE %	17.1 ± 6.8^c^	35.7 ± 13.3^a^	31.1 ± 27.2^b^

*F* *%*–mycorrhizal frequency, *M* *%* relative mycorrhizal root length, *m* *%* intensity of colonization within individual mycorrhizal root, *A* *%* arbuscule richness in the whole root system, *a* *%* arbuscule richness in root fragments where the arbuscules were present, *FDSE* *%* DSE frequency, *NS* not statistically significant

** p < 0.01, *** p < 0.001

The permutation tests based on RDAs yielded several significant variables which explained differences in AM and DSE colonization (Table 5). This analysis was almost congruent with comparison of community-level weighted means of trait values among groups. According to functional analysis non-mycorrhizal plants showed that this group is the most functional rich and is characterized by the lowest functional diversity. In turn, *Aster novi*-*belgii* group, comprising species with higher values of AM and DSE colonization, is the most functionally diverse followed by the second mycorrhizal group *Galinsoga ciliata* group in which values of functional analysis were a little lower (Table 5). This is an interesting result which demonstrates that species which are colonized by fungi are not homogeneous. Analysis of particular variables using RDA can shed more light on the question of which variables make species more vulnerable to fungi colonization. Competitive ability, height and no seed bank were features associated with non-mycorrhizal plants (Table 5). It was the influence of *Reynoutria* taxa which can grow up to 3 meters during vegetation season and do not form seed bank. Non-mycorrhizal species showed relatively high values of Ellenberg moisture index. Once again, taxa which are confined to moist habitats are *Reynoutria* taxa which are very invasive in river valleys (Gerber et al. 2008). Total invasiveness and number of habitats invaded as well as invading range in the country turned out to be significant. The species from *Aster novi*-*belgii* group which are most widely distributed also penetrate more types of habitats (Table 5). Mean higher residence time was found in *Galinsoga ciliata* group. Usually residence time is correlated with invading range and a species invasiveness (Pyšek and Jarošík 2005). In our case it could be biased by archaeophytes *Sonchus oleraceus* and *Avena fatua*. These species are post-invasive plants which were introduced a long time ago. We did not include all possible factors which are present in urban habitats. For instance we know very little about how urban conditions in terms of so called “urban heat island effect” (Bolund and Hunhammar 1999) influence AMF—plant interactions. The study area Katowice is a centre of conurbation which has population estimated at ca. 3.5 million. The effect of heat island also exist there what means that temperature can increase by 1.5 °C. Many urban thermophilous species e.g. *Eragrostis minor, Cardaria draba, Diplotaxis muralis* (Sudnik-Wójcikowska 2000) were either non-mycorrhizal or weakly colonized by AMF. Two of these species are non-invasive. It is not certain whether these plants are non-dependant on AMF or, on the contrary, whether biotopic conditions and initial phase of invasion do not enhance development of plant—AMF associations.

**Table 5:** The results of Monte Carlo test of Redundancy Analyses (RDA) based on AM and DSE colonization, functional analysis and selected explanatory variable and the community-level weighted means of trait values (CWM) among three groups of plants

Explanatory variable		CWM		
		Non-mycorrhizal species	Aster novi-belgii group	Galinsoga ciliata group
Plant traits	C/CSR competitive/intermediatestrategy**	**1**	**0**	**0**
	CR competive ruderal**	0	0	0
	R ruderal NS	0	0	0
	R/CR ruderal/competitive ruderal***	0	0	0
	SR stress ruderal***	**0**	**0**	**1**
	Height of plant*	**1.45**	**0.89**	**1.06**
	Long term persistent seed bank NS	0	0	0
	Short term persistent seed bank***	0	0	0
	Transient seed bank***	0	0	0
	No seed bank***	**1**	**0**	**0**
Habitat associations	F moisture**	6.2	5.25	5.43
	R soil reaction NS	6.8	3.25	5.14
	N trophy***	**6.4**	**7.25**	**5.71**
History of invasion in Poland	Range**	**3.4**	**3.88**	**4**
	Population size	3.8	4	3.43
	Habitats invaded**	**2**	**2.25**	**1.86**
	Tendency in spread	2.8	2.87	2
	Residence time**	**115.4**	**191.62**	**377.71**
	Total invasiveness*	**14.2**	**13.87**	**12.14**
Functional analysis	Functional richness	12.39	6.19	7.02
	Functional evenness	0.81	0.92	0.92
	Functional divergence	0.86	0.92	0.89
	Functional dispersion	3.94	4.16	4.09
	Rao’s quadratic entropy	16	17.5	17.14

The values which differ significantly are *bolded*

*NS* non-significant

* p < 0.05, ** p < 0.01, *** p < 0.001

## Conclusions

To summarise, we noticed that Asteraceae representatives, native to America, were characterized by both the highest values of AM and DSE colonization. In most of the studied species taxonomic pattern and AM colonization are significant factors in invasiveness and the taxa of the *Asteraceae* family are examples confirming this theory. On the other hand, taxa from the *Polygonaceae* family are also indicated as invasive but usually are non-mycorrhizal. Thus it can be inferred that taxonomic pattern better predicts species invasiveness than presence of AM. Moreover, many neophytes (including invasive species) in Central Europe originated from the temperate forest biome of eastern North America or eastern Asia (Chytrý, et al. 2005). The taxa of *Reynoutria* represent the contrary example of highly invasive plants compared to *Asteraceae* members. They are of Asiatic origin, non-mycorrhizal and weakly colonized by DSE fungi. It is not known if DSE colonization can enhance invasiveness of alien plant species because DSE were present in all studied species and in all samples.

To conclude, root endophytes can determine the success of non-native plant species in the process of plant invasion. However, it must be emphasized that for an alien plant species its distribution can be determined by the combination of certain abiotic and biotic variables, but because the group of plants in question is very heterogeneous, it is unlikely that a single hypothesis could explain their success of invasion.

## Electronic supplementary material

Below is the link to the electronic supplementary material.

Supplementary material 1 (DOC 48 kb)
