# Supplementary material for: AM and DSE colonization of invasive plants in urban habitat: a study of Upper Silesia (southern Poland)
Source: J Plant Res. 2016 Feb 19;129(4):603–14. doi: 10.1007/s10265-016-0802-7 (PMC4909803; doi:10.1007/s10265-016-0802-7)
Supplement: Supplementary file 1 — Supplementary material 1 (DOC 48 kb) [file 10265_2016_802_MOESM1_ESM.doc]

**Electric Supplementary Material**

**Title:**

AM and DSE colonization of invasive plants in urban habitat: a study of Upper Silesia (southern Poland)

**Authors:**

Gucwa-Przepióra Ewa, Chmura Damian, Sokołowska Kamila

**Journal:**

Journal of Plant Research

**Corresponding author:**

Ewa Gucwa-Przepióra

Department of Botany and Nature Protection,

Faculty of Biology and Environmental Protection,

University of Silesia,

Jagiellonska 28, PL 40-032 Katowice, Poland,

Phone: +48 32 2009366, Fax: +48 32 2009361

e-mail: [Ewa.Gucwa-Przepiora@us.edu.pl](mailto:Ewa.Gucwa-Przepiora@us.edu.pl), [egucwaprzepiora@gmail.com](mailto:egucwaprzepiora@gmail.com)

**Content:**

Table S1

**Table S1** A list of stands where plants were collected for mycorrhizal studies

|  | **Plant species** | **Localisation** | **GPS coordinates** | **Habitat** |
| --- | --- | --- | --- | --- |
| 1 | *Aster novi - belgii* | Katowice, ul. Kościuszki | 50°14'48.45"N 19°00'44.34"E | wasteland |
| 2 | *Avena fatua* | Katowice, ul. Powstańców | 50°15'07.65"N 19°01'19.24"E | roadside |
| 3 | *Bidens frondosa* | Tychy, ul. Bielska | 50°06'03.99"N 18°58'02.63"E | deciduous forest |
| 4 | *Cardaria draba* | Katowice, ul. Chorzowska | 50°16'04.67"N 19°00'37.99" E | roadside |
| 5 | *Conyza canadensis* | Katowice, ul. Podchorążych | 50°15'03.45"N 19°00'59.80"E | roadside |
| 6 | *Diplotaxis muralis* | Katowice, ul. Mikołowska | 50°15'06.49"N 19°00'23.39"E | roadside |
| 7 | *Echinocystis lobata* | Tychy, Paprocany | 50°05'26.25"N 18°59'08.05"E | wasteland |
| 8 | *Eragrosis minor* | Katowice, ul. Jagiellońska | 50°15'14.99"N 19°01'25.54"E | gaps between flagstones |
| 9 | *Erigeron annus* | Katowice, ul. Ceglana | 50°14'39.44"N 19°00'48.27"E | wasteland |
| 10 | *Galinsoga ciliata* | Katowice, ul. Królowej Jadwigi | 50°15'07.40"N 19°01'14.34"E | roadside |
| 11 | *Galinsoga parviflora* | Katowice, ul. Paderewskiego | 50°15'11.29"N 19°02'19.82"E | roadside |
| 12 | *Impatiens glandulifera* | Katowice, Muchowiec | 50°14'00.58"N 19°01'53.95"E | edge of the ditch |
| 13 | *Impatiens parviflora* | Katowice, Muchowiec | 50°14'00.58"N 19°01'53.95"E | mixed forest |
| 14 | *Reynoutria × bohemica* | Katowice, Muchowiec | 50°13'55.43"N 19.01'26.53"E | wasteland |
| 15 | *Reynoutria japonica* | Katowice, Muchowiec | 50°13'55.43"N 19.01'26.53"E | wasteland |
| 16 | *Reynoutria sachalinensis* | Katowice, Muchowiec | 50°13'55.43"N 19.01'26.53"E | wasteland |
| 17 | *Solidago canadensis* | Katowice, Kokociniec | 50°14'21.39"N 18°57'17.99"E | mixed forest, railway embankment |
| 18 | *Solidago gigantea* | Katowice, Panewniki | 50°13'31.14"N 18°56'38.07"E | wasteland |
| 19 | *Solidago graminifolia* | Katowice, Kokociniec | 50°14'21.39"N 18°57'17.99"E | mixed forest |
| 20 | *Sonchus oleraceus* | Katowice, ul. Przemysłowa | 50°15'15.39"N 19°01'58.24"E | roadside |
